# Adaptation Shapes Local Cortical Reactivity: From Bifurcation Diagram and Simulations to Human Physiological and Pathological Responses

**DOI:** 10.1523/ENEURO.0435-22.2023

**Published:** 2023-07-24

**Authors:** Anna Cattani, Andrea Galluzzi, Matteo Fecchio, Andrea Pigorini, Maurizio Mattia, Marcello Massimini

**Affiliations:** 1 Department of Mathematics and Statistics, Boston University, Boston, MA 02215; 2 National Center for Radiation Protection and Computational Physics, Istituto Superiore di Sanità, Rome 00161, Italy; 3 Center for Neurotechnology and Neurorecovery, Department of Neurology, Massachusetts General Hospital and Harvard Medical School, Boston, MA 02114; 4 Department of Biomedical, Surgical and Dental Sciences, Università degli Studi di Milano, Milan 20122, Italy; 5 Department of Biomedical and Clinical Sciences “L. Sacco,” Università degli Studi di Milano, Milan 20157, Italy; 6 Istituto Di Ricovero e Cura a Carattere Scientifico, Fondazione Don Carlo Gnocchi, Milan 20122, Italy; 7 Azrieli Program in Brain, Mind and Consciousness, Canadian Institute for Advanced Research (CIFAR), Toronto, Ontario M5G 1M1, Canada

**Keywords:** bifurcation theory, cortical bistability, cortical network model, off-period

## Abstract

Human studies employing intracerebral and transcranial perturbations suggest that the input-output properties of cortical circuits are dramatically affected during sleep in healthy subjects as well as in awake patients with multifocal and focal brain injury. In all these conditions, cortical circuits react to direct stimulation with an initial activation followed by suppression of activity (Off-period) that disrupts the build-up of sustained causal interactions typically observed in healthy wakefulness. The transition to this stereotypical response has important clinical implications, being associated with loss of consciousness or loss of functions. Here, we provide a mechanistic explanation of these findings by means of simulations of a cortical-like module endowed with activity-dependent adaptation and mean-field theory. First, we show that fundamental aspects of the local responses elicited in humans by direct cortical stimulation can be replicated by systematically varying the relationships between adaptation strength and excitation level in the network. Then, we reveal a region in the adaptation-excitation parameter space of crucial relevance for both physiological and pathologic conditions, where spontaneous activity and responses to perturbation diverge in their ability to reveal Off-periods. Finally, we substantiate through simulations of connected cortical-like modules the role of adaptation mechanisms in preventing cortical neurons from engaging in reciprocal causal interactions, as suggested by empirical studies. These modeling results provide a general theoretical framework and a mechanistic interpretation for a body of neurophysiological measurements that bears critical relevance for physiological states as well as for the assessment and rehabilitation of brain-injured patients.

## Significance Statement

Suppression of cortical activity following an initial activation is a defining feature of deep sleep in healthy subjects and wakefulness in patients affected by focal and multifocal brain injuries. Experimental findings suggest that these bimodal responses disrupt the emergence of complex interactions among cortical regions, leading to loss of consciousness or functional impairments. Given their practical implications, studying the mechanisms involved within a general theoretical framework is essential. Using a neuronal network model, we provide evidence for the key role of activity-dependent adaptation mechanisms in shaping the responses to perturbation and affecting the build-up of complex cortical interactions. Overall, this work provides a mechanistic interpretation relevant to the stratification, follow-up, and rehabilitation of brain-injured patients.

## Introduction

Studies combining transcranial magnetic stimulation (TMS) with electroencephalographic (EEG) recordings have demonstrated a dramatic impairment of the capacity of cortical networks to engage in large-scale interactions during sleep and anesthesia (Massimini et al., 2005; Ferrarelli et al., 2010; Sarasso et al., 2015): while during wakefulness the initial cortical activation triggers a chain of recurrent waves of activity and a long-range, complex pattern of interactions (Casali et al., 2013; Comolatti et al., 2019), during both non-rapid eye movement (NREM) sleep and anesthesia such distributed, rich spatiotemporal activity is lost and replaced by a response that is simple and stereotypical. Intracranial explorations employing single-pulse intracortical electrical stimulations and local field potential recordings in humans (Pigorini et al., 2015; Usami et al., 2015) showed that this stereotypical response consists of an initial activation rapidly followed by silencing of neuronal firing (Off-period), as assessed by a significant suppression of high-frequency activity (Mukovski et al., 2007; Cash et al., 2009). This tendency for cortical circuits to fall into an Off-period after initial activation during sleep and anesthesia, also known as cortical bistability (Tononi and Massimini, 2008; Nir et al., 2011), is in a key position to prevent the emergence of the complex interactions observed during wakefulness. Indeed, Off-periods not only do temporarily interrupt but also disrupt neuronal activity: as demonstrated by phase locking analysis, when neuronal activity resumes after each Off-period, it does so in a stochastic manner retaining no causal relationship with the initial input.

Crucially, this dramatic change in the input-output properties of cortical circuits is not only typical of physiological sleep and anesthesia but can also occur during wakefulness in pathologic conditions. Accumulating evidence shows that cortical circuits react to perturbations with an Off-period also in awake unresponsive wakefulness syndrome patients (UWS) (Rosanova et al., 2018), previously known as vegetative state patients, as well as in the perilesional area surrounding focal cortical lesions in awake stroke patients (Sarasso et al., 2020; Tscherpel et al., 2020) with profound clinical implications. When cortical bistability involves most of the cortex, such as in the UWS, large-scale brain interaction collapse leading to loss of consciousness (Rosanova et al., 2018). When cortical bistability is local, such as that found in the perilesional areas of stroke patients, it leads to regional circuit impairment and selective functional deficits (Sarasso et al., 2020; Tscherpel et al., 2020).

Given the clinical relevance of these findings, it is crucial to understand the nature of evoked Off-periods and their impact on cortical responsiveness within a mechanistic framework. An interesting hypothesis is that adaptation mechanisms play an important role in generating the Off-periods observed after cortical stimulation. Activity-dependent adaptation accounts for local fatigue mechanisms, i.e., self-inhibition, which lowers the firing rate of neuronal populations. At the level of cortical circuits, adaptation can be induced by several microscopic biophysical mechanisms involving calcium-dependent potassium channels, short-term synaptic depression (Gerstner et al., 2014) and/or GABAergic synaptic transmission (Sanchez-Vives et al., 2021). As suggested by mean-field theories, activity-dependent adaptation is involved in the generation of the rhythmic alternation between On-periods (high-activity up state) and Off-periods (low-activity down state) spontaneously occurring during slow-wave sleep (Latham et al., 2000; Gigante et al., 2007).

Here, we ask whether activity-dependent adaptation can reproduce and explain the fundamental features of the alteration of cortical responsiveness observed empirically during physiological sleep and during wakefulness in pathologic conditions. We address this question within a general formal framework resting on bifurcation analysis and in-silico simulations of cortical modules endowed with activity-dependent adaptation (Mattia and Sanchez-Vives, 2012). We show how such a model can provide a parsimonious explanation for the three key features characterizing the alteration of cortical reactivity observed in humans. First, using bifurcation analysis of the model we classify its dynamical regimes and reproduce parametrically both spontaneous and stimulus-evoked dynamics empirically observed during sleep and pathologic wakefulness. Second, using the same framework, we explain the empirical finding that direct cortical stimulation is more effective than the observation of ongoing dynamics in revealing Off-periods in both physiological and pathologic conditions. Third, by considering two linked modules we show how changing the adaptation level alone results in a break-off of reciprocal corticocortical interactions as observed *in vivo*.

## Materials and Methods

### Spiking neuron network

**Figure 1.**
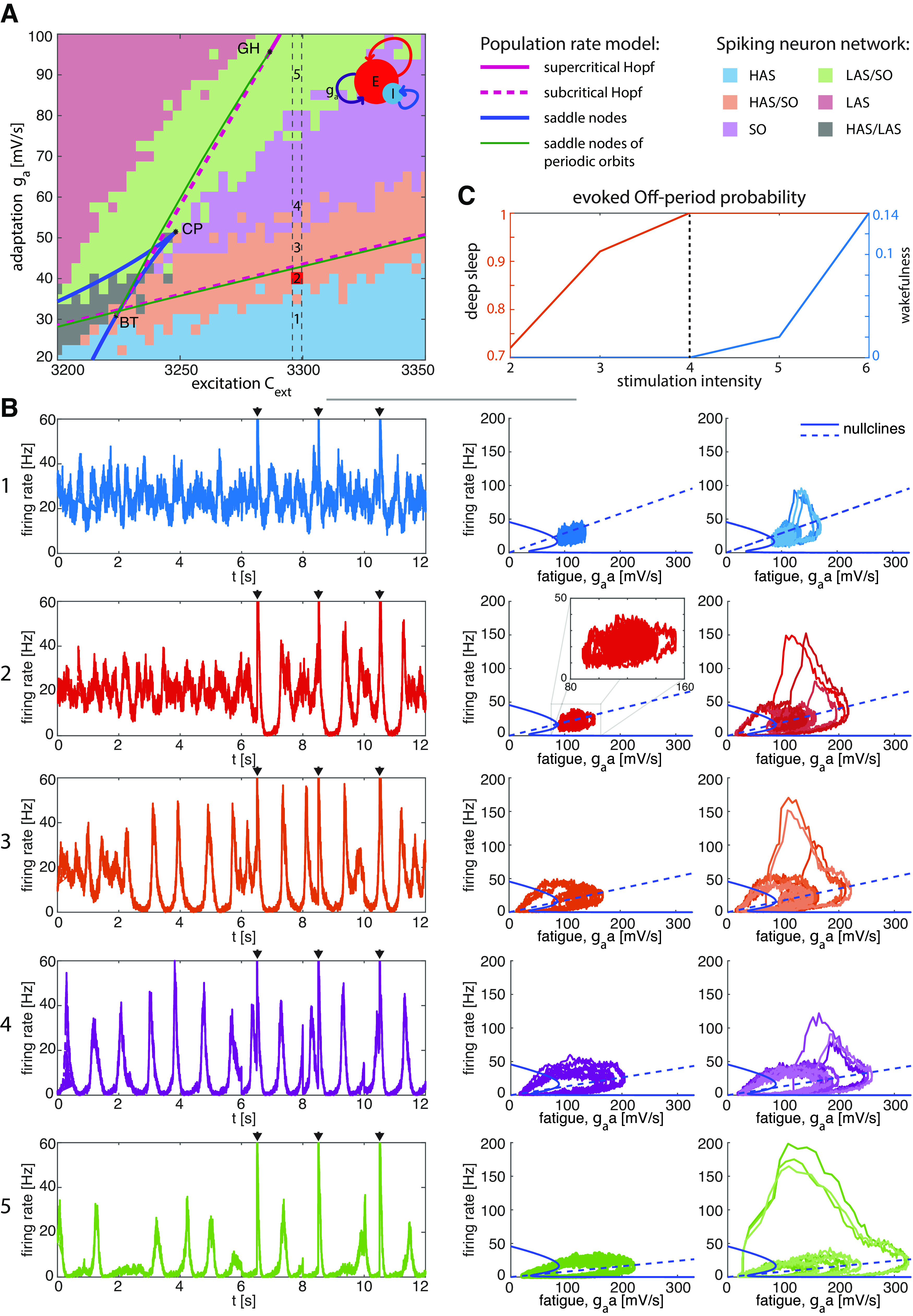
Dynamical regimes of the spiking neuron network accompanied by the bifurcation analysis of the population rate model. ***A***, Dynamical regimes of the spiking neuron network in the excitation-adaptation parameter space, namely ( $C_{ext},g_{a}$), classified according to its spontaneous activity (for details, see Materials and Methods, Analysis of the dynamical regimes of the spiking neuron network) superimposed to the codimension-two bifurcation diagram of the population rate model. The codimension-one bifurcations encompass the subcritical Andronov–Hopf bifurcation curves (dashed magenta lines), the supercritical Andronov–Hopf bifurcation curves (solid magenta line), the saddle-node bifurcation curve (blue curves), and the limit point of cycles (green lines, also known as saddle nodes of periodic orbits) that turn the periodic orbits originating from Andronov–Hopf stable. The codimension-two bifurcation Bogdanov–Takens (BT) is the contact point between the saddle-node bifurcation curve and Andronov–Hopf bifurcation curves. The saddle-node curve shows a cusp bifurcation (CP). The areas among the curves have been color-coded according to the regime shown by the spontaneous dynamics of the spiking neuron network. The five different dynamical regimes are characterized by HAS (high-asynchronous state; blue area), HAS/SO (high-asynchronous state with incursions of slow oscillations, namely with incursions of Off-periods because of finite-size effects; orange area), SO (slow oscillations; purple area), LAS/SO (low-asynchronous state with incursions of slow oscillations; green area), and, finally, LAS (low-asynchronous state; green area). A sixth region (gray area), not of interest in the present work, is characterized by HAS and LAS alternating at an irregular pace. ***B***, Spontaneous and stimulus-evoked signals with the same color-coding used for the dynamical regimes of the spiking neuron network (panel ***A***) for a fixed excitation ( $C_{ext}=3297.5)$ and several adaptation levels [Case 1: 30; Case 2: 40; Case 3: 45; Case 4: 57.5; Case 5: 90 (mV/s)]. Left column, Time series encompassing both spontaneous activity (up to 6000 time steps) and stimulus-evoked activity (remaining interval). Black triangles indicate the occurrence of the stimulation. Central column, Spontaneous activity as a function of fatigue ( $g_{a}\cdot a$ mV/s). Right column, Three superimposed orbits because of perturbations as a function of the fatigue. In both the central and right columns, the spontaneous and stimulus-evoked dynamics are superimposed to the nullclines of the population rate model. The intersection of the nullclines represents the equilibrium point of the corresponding population rate model. The equilibria of the spiking neuron network and the population rate model show a close albeit not complete overlap. The dynamics of the spiking neuron network for a fixed excitation and different adaptation levels are provided in Extended Data Figure 1-1. ***C***, Probability to evoke Off-periods in wakefulness (Case 1) and deep sleep (Case 3) as a function of several stimulation intensities spanning from 2 to 6 ([a.u.]). The probability to evoke Off-periods is computed as the number of trials, lasting 2 s each, showing at least one Off-period divided by the total number of trials (50).

The neuronal network model is adapted from (Torao-Angosto et al., 2021), which has been proven to quantitatively reproduce the statistical features of the spontaneous up-down slow oscillations recorded in Layer 5 of the visual cortex of sleeping and anesthetized rats. Briefly, the network is composed of 6300 excitatory (E) and 2580 inhibitory leaky integrate-and-fire (LIF) neurons. The membrane potential $V_{i}$ of the i-th neuron evolves as

(1) $${\dot{V}}_{i}=-\frac{V_{i}}{\tau }+I_{i}-g_{a}a_{i}$$

$${\dot{a}}_{i}=-\frac{a_{i}}{\tau_{a}}+\underset{k}{\sum }\delta (t-t_{ik}),$$

emitting its k-th spike at time $t_{ik}$ if $V_{i}\left(t_{ik}\right)\geq V_{thr}$ for the first time from the emission of its previous action potential. Following the spike emission, $V_{i}=V_{res}$ for a refractory absolute period $\tau_{0}$ [2 ms (1 ms) for E (I) neurons] before restarting its time evolution (Eq. 1). Reset potential $V_{res}=15$ mV and the emission threshold $V_{thr}=20$ mV are the same for both neuron types, while decay constant τ is 20 and 10 ms for E and I neurons, respectively. The synaptic current $I_{i}\left(t\right)=\overset{N}{\underset{j=1}{\sum }}J_{ij}\overset{}{\underset{k}{\sum }}\delta \left(t-t_{jk}-d_{ij}\right)+J_{ext}\overset{}{\underset{k}{\sum }}\delta (t-t_{ext,k})$ results from the spiking activity of the presynaptic neurons mediated by recurrent synaptic efficacies $J_{ij}$ randomly selected to be different from zero with connection probability $c_{EE},c_{EI},c_{IE},c_{II}=\{0.6,5,0.2,1.7\}\%$. $J_{ij}$ takes positive or negative values according to the type of the j-th presynaptic neuron, and it is randomly extracted from a truncated Gaussian distribution with mean $J_{EE},J_{EI},J_{IE},J_{II}=\left\{1.9,-1.1,2.2,-1.1\right\}$ mV and a relative standard deviation of 25%. Presynaptic spikes are delivered with an axonal delay $d_{ij}$ randomly sampled from an exponential distribution with an average of 22.6 and 5.7 ms for E and I presynaptic neurons, respectively, modeling noninstantaneous synaptic transmission (Mattia et al., 2019). External neurons contribute to $I_{i}\left(t\right)$ at baseline as a Poissonian spike train made of $C_{ext}$ independent sources each with an average firing rate of $\nu_{ext}=0.25$ Hz. Here, for E neurons, $C_{ext}$ range from 3200 and 3350 in the bifurcation diagram of Figure 1, while $C_{ext}=733$ for I neurons. The external stimulations consist of increasing the frequency $\nu_{ext}$ by a factor 4 (i.e., the stimulation intensity) for a short period of 2 ms. The external k-th spike occurring at $t_{ext,k}$ affects $I_{i}\left(t\right)$ with efficacy $J_{ext}=$ 0.48 and 2.2 mV for E and I neurons, respectively.

10.1523/ENEURO.0435-22.2023.f1-1 Extended Data Figure 1-1 Bifurcation analysis of the population rate model and dynamical regimes of the spiking neuron network (as in Fig. 1) for a fixed adaptation level ($g_{a}=42.5$ mV/s) and different excitation levels (3342.5, 3320, 3248.75). ***A***, ***C***, As in Figures 1 and 3, respectively. In panel ***C***, the differences between the probability of evoked Off-periods and spontaneous Off-periods for Cases 1–3 are as follows: Case 1: 0.08; Case 2: 0.46; Case 3: 0.14. ***B***, Spontaneous and stimulus-evoked signals (using the same color coding as in ***A***) for a fixed level of adaptation. Left column, Time series encompassing both spontaneous activity (up to 6000 time steps) and stimulus-evoked activity (remaining interval). Black triangles indicate the occurrence of the stimulation. Central column, Spontaneous activity as a function of fatigue. Right column, Three superimposed orbits due to perturbations as a function of the fatigue. Download Figure 1-1, TIF file.

The second equation in Equation 1 provides the description of the activity-dependent fatigue mechanism modeling the spike-frequency adaptation (Benda and Herz, 2003; Gigante et al., 2007; Mattia and Sanchez-Vives, 2012) that affects the excitatory neurons. Specifically, the dynamics of the variable $a_{i}\left(t\right)$ is defined by the spiking activity of the excitatory neurons: at each spike, the adaptation $a_{i}\left(t\right)$ undergoes a unitary increase and relaxes exponentially until the next spike is emitted. Thus, the higher the excitatory population firing rate, the higher the variable $a_{i}\left(t\right)$. However, how much the $a_{i}\left(t\right)$ dynamic modulates the excitatory population firing rate is also determined by the adaptation level $g_{a}$ ranging from 20 to 100 mV/s and the relaxation time constant for the adaptation level set at $\tau_{a}=150$ ms. In general, the higher the $a_{i}\left(t\right)$ because of the spikes emitted by the i-th neuron and adaptation level $g_{a}$, the higher the excitatory population self-inhibition (eventually reducing its firing rate). This fatigue mechanism mimics the extracellular ionic concentrations (calcium and/or sodium) that drive a hyperpolarizing potassium current. The choice to model only the activity-dependent adaptation associated with the calcium-dependent potassium currents described above does not affect the generality of the result we presented. Indeed, under the mean-field approximation detailed in the next section, the nonlinear dynamics of the network firing rate governed by other forms of activity adaptation are qualitatively similar. More specifically, the phase-plane analysis of spiking neuron networks incorporating adaptation mechanisms like short-term synaptic depression (Holcman and Tsodyks, 2006) and slow GABAergic inhibition (Parga and Abbott, 2007) are known to display similar nullclines and equilibrium states.

We also performed simulations of a network made of two identical modules. Each module is made by E-neurons and I-neurons described by Equation 1 with all the parameters set as in the single module, with the exception of the relative standard deviation for synaptic efficacies. Since the relative standard deviation for synaptic efficacies is set to zero in the two-module case, synaptic efficacies are $J_{EE},J_{EI},J_{IE},J_{II}\;=\left\{1.9,-1.1,2.2,-1.1\right\}$ mV. Furthermore, the two modules interact with each other through connections established between excitatory neurons only, with synaptic efficacy $J_{12}=J_{21}\;$ set to 1.18 mV, where 1 and 2 identify the two modules. The probability of connection $c_{12},c_{21}$ for each excitatory neuron is 0.1%, and average axonal delays are set to $d_{12}=55$ ms for connections from the first to the second module, and $d_{21}=50$ ms from the second to the first module. Furthermore, we considered the modules in a low adaptation regime, where both the modules are characterized by $g_{a}=48$ mV/s, and high adaptation regime, where $g_{a}=78$ mV/s.

Simulations of the spiking neuron network were performed with an open-source program relying on an event-driven numerical integration described in (Mattia and Del Giudice, 2000). To run this software with the appropriate parameters, we used a MATLAB code available at https://github.com/annacatt/Adaptation_and_cortical_responses and available as Extended Data 1.

### Population rate model

Under mean-field approximations requiring many presynaptic contacts $c_{\alpha \beta }N$ and limited firing rates $\nu_{\alpha }$ (Amit and Tsodyks, 1991; Amit and Brunel, 1997), where α and β identify different interacting populations, the evolution of the instantaneous firing rate $\nu_{\alpha }(t)$ of an infinite-size network is described by $\tau_{\nu }\overset{\nu_{\alpha }}{˙}=-\nu_{\alpha }+\Phi (\nu_{\alpha })$ (Treves, 1993; Brunel and Hakim, 1999; Mattia and Del Giudice, 2002). Here, the current-to-rate gain function Φ is the Siegert–Ricciardi one (Siegert, 1951; Capocelli and Ricciardi, 1971):

$$\Phi \left(\mu ,\sigma \right)={\left[\tau_{0}+\tau \sqrt{\pi }\overset{(V_{thr}-\mu \tau )/\sqrt{\sigma^{2}\tau }}{\underset{(V_{res}-\mu \tau )/\sqrt{\sigma^{2}\tau }}{\int }}\left[1+\text{erf}\left(z\right)\right]e^{z^{2}}dz\right]}^{-1},$$

where μ and $\sigma^{2}$ are, respectively, the infinitesimal mean and variance of the input current (Amit and Tsodyks, 1991; Amit and Brunel, 1997) whose form is shown below, and erf is the error function. The first-order dynamical equation $\tau_{\nu }{\dot{\nu }}_{\alpha }=-\nu_{\alpha }+\Phi (\nu_{\alpha })$ can be seen as the simplest relaxation dynamics leading to the asymptotic firing rates given by $\Phi (\nu_{\alpha })$. Here, $1/\Phi (\nu_{\alpha })$ is the mean of the interspike intervals (ISI) expected for the leaky integrate-and-fire neuron driven by a stochastic current with infinitesimal mean μ and variance $\sigma^{2}$. The ISI in this model is the time when the membrane potential V(t) crosses the emission threshold $V_{thr}$ for the first time starting from the reset potential $V_{res}$ at time t=0. This interval also includes the absolute refractory period ( $\tau_{0}$). The above dynamics for $\nu_{\alpha }(t)$ is obtained by resorting to a spectral expansion of the Fokker–Planck equation and determining the evolution of the probability density of the membrane potential. The first-order ordinary differential equation results from taking into account only the slowest eigenmode, i.e., the eigenfunctions of the Fokker–Planck operator with the highest eigenvalue (Treves, 1993; Mattia and Del Giudice, 2002). This first-order ODE recovers the phenomenological dynamics for the firing rate introduced by Wilson and Cowan (1972). Since the cortical module we are considering includes two interacting populations of excitatory and inhibitory neurons, the mean-field dynamics is:

$$\tau_{E}{\dot{\nu }}_{E}=-\nu_{E}+\Phi_{E}\left(\mu_{E},\sigma_{E}\right)$$

(2) $$\tau_{I}{\dot{\nu }}_{I}=-\nu_{I}+\Phi_{I}(\mu_{I},\sigma_{I})$$

$$\dot{a}=-a/\tau_{a}+\nu_{E},$$

where α∈{E,I}, $\Phi_{\alpha }(\mu_{\alpha },\sigma_{\alpha })$ is the above gain function computed with the single-neuron parameters of type α and $\tau_{\alpha }$ is the decay constant of the LIF membrane potential. The infinitesimal mean and variance of the input current are defined as:

$$\mu_{\alpha }=c_{\alpha E}N_{E}J_{\alpha E}\nu_{E}+c_{\alpha I}N_{I}J_{\alpha I}\nu_{I}+C_{\alpha ,ext}J_{\alpha ,ext}\nu_{ext}-g_{\alpha }a$$

$$\sigma_{\alpha }^{2}=c_{\alpha E}N_{E}J_{\alpha E}^{2}(1+\Delta_{\alpha E}^{2})\nu_{E}+c_{\alpha I}N_{I}J_{\alpha I}^{2}(1+\Delta_{\alpha I}^{2})\nu_{I}+C_{\alpha ,ext}J_{\alpha ,ext}^{2}(1+\Delta_{\alpha ,ext}^{2})\nu_{ext},$$

with α,β∈{E,I} and $\Delta_{\alpha \beta }$ are the relative standard deviations of the synaptic efficacies $J_{ij}$ for the different neuronal types. This mean-field approximation effectively describes the collective dynamics of networks of LIF neurons with spike-frequency adaptation even far from the equilibrium (Gigante et al., 2007; Mattia and Sanchez-Vives, 2012). Here, only excitatory neurons have adaptation: $g_{E}\equiv g_{a}$ and $g_{I}=0$.

Finally, because of the breaking of the diffusion approximation valid only in the limit $J_{\alpha \beta }\to 0$, we shifted horizontally the critical point of this bifurcation diagram by an appropriate amount ( $\Delta C_{E,ext}=-310$) of excitation level. Indeed, in the diffusion limit the currents received by the neurons are continuous stochastic processes determined by the continuous barrage of the synaptic input arriving at high rates and inducing small jumps in the membrane potential (Tuckwell, 1988). However, in the model network we simulated, synaptic efficacies are not negligible and the membrane potentials have a jump-like evolution in time. In this shot-noise regime, firing rates are lower compared with the ones predicted under the diffusion approximation (Richardson and Swarbrick, 2010), and this explains why we need to incorporate this effect by reducing the excitation level in our mean-field theory, eventually leading to a rightward shift of the theoretical bifurcation diagram. Furthermore, we also shifted vertically the critical points of the bifurcation diagram by an appropriate amount ( $\Delta g_{a}=-7$ mV/s) of adaptation. Besides the breaking of the diffusion approximation, the shifts $\Delta C_{E,ext}$ and $\Delta g_{a}$ are expected to be theoretically justified by the fact that the simulated networks are composed of a finite number of spiking neurons challenging the infinite-size limit on which extended mean-field approximation relies on (Mattia and Del Giudice, 2002).

### Simulated data and data preprocessing

Simulations consist of *N* trials (*N* = 50 for the single cortical module and *N* = 250 for the case of two interacting modules). The external stimulus in each trial was delivered at time *t* = 0, with a prestimulus interval lasting randomly between 5000 and 5300 ms and poststimulus interval lasting 5000 ms. Stimulation artifact was reduced by applying a Tukey-windowed median filtering, as in (Chang et al., 2012), between −5 and 5 ms (Pigorini et al., 2015).

### Bifurcation analysis of the population rate model

A detailed analysis of local bifurcations of Equation 2 was performed through MatCont (Dhooge et al., 2003), a MATLAB Toolbox that allows computing curves of equilibria and bifurcation points through a prediction-correction continuation algorithm, as described previously (Kuznetsov, 2004).

### Analysis of the dynamical regimes of the spiking neuron network

The dynamical regimes of the spiking neuron network (Eq. 1) were determined according to the frequency of On-periods and Off-periods (up and down states, respectively) detected across 50 trials of spontaneous activity lasting 2 s each. The detection of the On-periods and Off-periods was conducted by looking at the firing rate in each trial. If the firing rate exceeded 20 Hz, the trial contained at least one On-period. On the contrary, if the firing rate fell under 5 Hz, the trial contained at least one Off-period. Once we collected the probability of On-periods and Off-periods across 50 trials for each value of excitation and adaptation level, we classified five dynamical regimes of interest. Following nomenclature previously published (Gigante et al., 2007; Mattia and Sanchez-Vives, 2012), high-asynchronous state (HAS) is characterized by a probability of On-periods equal to 1 (i.e., sustained high firing rate in every trial) and absence of Off-periods. Low-asynchronous state (LAS) presents a probability of Off-periods equal to 1 (i.e., persistent low firing rates in every trial) and absent On-periods. Slow oscillations state (SO) is characterized by both probabilities of On-periods and Off-periods equal to 1 since each trial sees On-periods and Off-periods alternating at a regular pace. Furthermore, on the border between LAS and SO, there is a region where the probability of Off-periods is equal to 1 with incursions of On-periods states (non-zero probability of On-periods < 1). Vice versa, the region between HAS and SO is characterized by On-periods in all the trials with incursions of Off-periods (non-zero probability of Off-periods <1). Finally, a sixth region, which is not the focus of this work, encompasses both HAS and LAS with non-zero probability of On-periods and Off-periods lower than 1. This is the case of heterogeneous trials, some containing only On-periods, some only Off-periods, and some exhibiting both On-periods and Off-periods alternating at an irregular pace.

### Simulated data analysis in the power and phase domain

Data analysis was performed using MATLAB R2020b (The MathWorks Inc.). We used the *timef* function implemented in EEGLAB (Delorme and Makeig, 2004) to detect transient event-related spectral perturbation (ERSP) and event-related phase-locking, i.e., inter-trial coherence (ITC) events, in the simulated data. More in detail, single trials were time-frequency decomposed between 5 Hz and 45 Hz using Wavelet transform (Morlet, window span: 3.5 cycles). The resulting ERSPs and ITCs were averaged across trials and normalized by subtracting the mean spectral activity of the prestimulus baseline from −1000 to −400 ms (from −5000 to −400 ms in the two-module network). To detect statistically significant activation with respect to the prestimulus baseline, we applied bootstrap statistics to both ERSP and ITC with significance level α = 0.005 and 1000 permutations. Nonsignificant bins were zeroed out. Furthermore, to discard the spurious intertrial coherent events associated with the absence of firing rate activity underlying the ERSP suppression, we retained only the ITC points associated with a significant increase in the ERSP power with respect to the prestimulus baseline. Finally, we averaged the ITC values across frequencies for each time point. The resulting average ITC provides an indication of the duration of the deterministic effect in a wide frequency window of a given external input. The code for the analysis is available at https://github.com/annacatt/Adaptation_and_cortical_responses and as Extended Data 1.

Methods and results related to human data are presented in published articles (Massimini et al., 2007; Pigorini et al., 2015; Rosanova et al., 2018; Sarasso et al., 2020) cited throughout this work and derive from analysis in the power and phase domains similar to the ones used for the simulated data presented here.

### Code accessibility

The code created for this paper is freely available online at https://github.com/annacatt/Adaptation_and_cortical_responses and is available as Extended Data 1.

### Data availability

All the simulated data that support the findings of this study are available online at https://figshare.com/articles/dataset/Simulated_data/21112603.

## Results

We employ a spiking network model to investigate to what extent varying the strength of adaptation and excitation can explain fundamental dynamics of cortical responsiveness empirically observed in healthy humans and brain-injured patients. Specifically, we aim to replicate and provide a mechanistic explanation of experimental data encompassing TMS-EEG recordings in healthy subjects during NREM sleep stage 2 (N2) (Massimini et al., 2007), in UWS patients with severe brain injury (Rosanova et al., 2018), in stroke patients (Sarasso et al., 2020; Tscherpel et al., 2020), as well as intracranially-evoked potentials in wakefulness/sleep (Pigorini et al., 2015).

### Bifurcation analysis and characterization of the dynamical regimes

Activity-dependent adaptation and excitation level shape the ongoing and stimulus-evoked activity of the simulated cortical module (Eq. 1). To fully characterize the dynamical regimes the model accounts for, we first performed a bifurcation analysis of its continuous counterpart (Eq. 2) exploiting a numerical continuation technique (Dhooge et al., 2003; Kuznetsov, 2004). This analysis expands on previous studies (Gigante et al., 2007; Mattia and Sanchez-Vives, 2012), providing a complete characterization of the bifurcations of the mean-field dynamics of the network. Taking into account only two key parameters, i.e., the level of excitation ( $C_{ext}$) and adaptation ( $g_{a}$; see Materials and Methods), the bifurcation diagram in Figure 1*A* shows critical points such as saddle nodes (blue traces) and subcritical Hopf bifurcations (dashed magenta traces). Subcritical Hopf bifurcations lie near the saddle nodes of periodic orbits (green traces), also known as limit points of cycles, where stable limit cycles arise. Thus, in the region limited by these two curves, stable slow oscillations are generated, corresponding to the cortical bistability observed in neurophysiological recordings performed in anesthetized rats (Tort-Colet et al., 2021). On the contrary, the region enclosed by the limit point curve (blue trace) is characterized by two stable and one unstable equilibrium solutions. The coexisting stable equilibria are characterized by high and low asynchronous firing states. In order to disambiguate, it is worth noting that this regime, which is not the focus of the present work, is also indicated with the term “bistable” as classically understood in the field of dynamical systems (Gerstner et al., 2014).

To check the effectiveness of this bifurcation diagram, we characterized the spontaneous dynamical regimes of equivalent networks of spiking (adaptive leaky integrate-and-fire) neurons in Equation 1. The leftmost part of the traces in Figure 1*B*, left column, shows 6 s of spontaneous activity for a given excitation value and five different adaptation levels. By taking into account the probability of Off-periods and On-periods during spontaneous activity (for details, see Materials and Methods), we identified different regions. The light blue region displays HAS (high-asynchronous state), i.e., relatively high firing rate and absence of Off-periods, which is typical of wakefulness. The light red region is characterized by LAS (low-asynchronous state), i.e., low asynchronous firing rate distinctive of Off-periods and absence of On-periods, a pattern resembling burst-suppression. The purple region features a regular alternation between HAS and LAS at a regular pace generating slow oscillations (SOs), as usually observed under anesthesia. The transition between these regimes is not as sudden as in the population rate model while varying one or both parameters. Indeed, finite-size effects make a state dominant, with incursions of the state of the nearby region (Gigante et al., 2007; Mattia and Sanchez-Vives, 2012). This is the case of the orange and green regions characterized by HAS and LAS, respectively, with incursions of SO. Specifically, HAS sparsely interspacing SO well describes deep sleep recordings.

### Perturbations reveal cortical bistability beyond spontaneous activity

Crucially, we further characterized the dynamical regimes of the spiking neuron model (Eq. 1) by exploring its responses to perturbations, which consist of an external current lasting 2 ms with intensity 4 ([a.u.]) injected into the excitatory neurons resembling the effects of direct magnetic and electrical stimulation. The role of the perturbation is to transiently alter the spontaneous population firing rate, potentially unveiling interesting activity-dependent properties of the system. The rightmost part of the traces in Figure 1*B*, left column (presenting Cases 1–5) shows 6 s of the stimulus-evoked activity for a given excitation value and five different adaptation levels. In the middle and right columns, the firing rate traces have been represented as a function of the average fatigue determining the adaptation of spike rates for both the spontaneous and the stimulus-evoked activity. The external stimulus applied to the network (defined by duration and intensity) increases the magnitude of the external Poisson spike trains. We fixed the duration to 2 ms and checked for the presence of Off-periods in the evoked activity for several stimulus intensities. As shown in Figure 1*C*, the stimulus intensity was finally set to never (always) evoke Off-periods in Case 1 (Case 3), which describes wakefulness (deep sleep).

**Figure 2.**
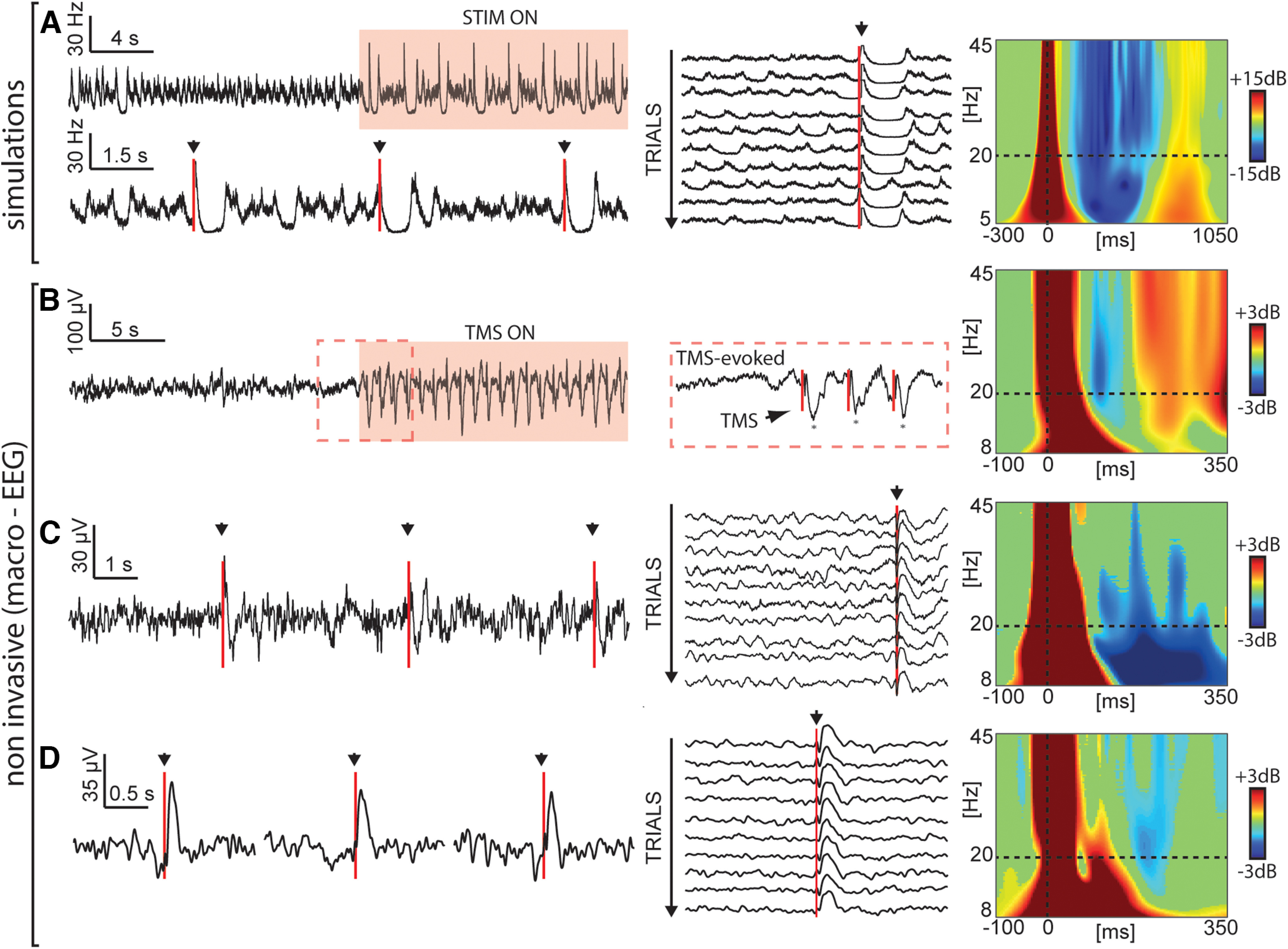
Perturbations trigger an Off-period regardless of spontaneous baseline activity in both simulated data, with adaptation level sets as in Case 2 of Figure 1, and in TMS-EEG data (during physiological N2 sleep, UWS patients, and in the perilesional area of stroke patients). First column, Time series with a few stimulations. Second column, Trials without Off-periods in the baseline aligned to the stimulation onset. Third column, Event-related spectral perturbation (ERSP). Blue color indicates a significant reduction compared with the baseline, while red indicates a significant increase. Black triangles and red lines indicate the occurrence of the stimulation. ***A***, Simulated firing rate data. ***B***, Re-edited from Massimini et al. (2007). EEG signal recorded from a channel (Cz) located under the stimulator during one TMS-ON block over a background of spontaneous NREM sleep (single-subject data). The TMS-ON block consisted of 40 stimuli at 0.8 Hz. The red dashed section shows the slow waves triggered at the beginning of the block. ***C***, Re-edited from (Rosanova et al., 2018). EEG recordings during TMS stimulations in a UWS patient. EEG activity of one representative electrode, Cz, while TMS was delivered with an interstimulus interval randomly jittering between 5000 and 5300 ms. Middle panel, Trials aligned to the stimulation displaying baseline activity without spontaneous slow waves. Panel ***D*** is derived from published data presented in Table 1 and Figure 2 in Sarasso et al. (2020). EEG recordings during perilesional TMS stimulations in a middle cerebral artery ischemia patient (patient 4). EEG activity recorded from a channel (Fc3) located over the perilesional area while TMS was delivered with an interstimulus interval randomly jittering between 2000 and 2300 ms. ERSPs in ***B–D*** were computed as by Rosanova et al. (2018).

In Case 1, the stimulus-evoked activity and spontaneous activity do not show relevant dissociations, as both are characterized by the absence of Off-periods, as empirically observed during wakefulness in human data. Higher adaptation levels, corresponding to Cases 3–5, are all characterized by the presence of Off-periods in both the spontaneous and evoked activity. The overall time the system spends in the Off-periods increases with the adaptation level: (1) Case 3 shows Off-periods interspersed with up states, thus resembling NREM sleep stage 3 (N3); (2) Case 4 displays regular alternation between On-periods and Off-periods, thus mirroring the effect of anesthesia on brain activity; (3) Case 5 reminds of burst suppression characterized by prolonged Off-periods. Crucially, Case 2 in Figure 1*B* reveals a clear-cut dissociation between the dynamical features observed in the spontaneous activity and the stimulus-evoked response. Here, while Off-periods are extremely rare (one every ∼16 s on average) in spontaneous activity, they can be reliably triggered by 76% of the stimulations. Hence, the model shows the existence of a particular regime whereby an interventional approach manifests a substantial sensitivity in revealing the underlying state of the system and its position in the bifurcation graph compared with an observational approach. In other words, it provides a crucial tool to detect cortical bistability with a few stimulations instead of relying on long spontaneous recordings. Such dissociation between observable dynamics and responses to perturbations is important because it reproduces a key feature reported by empirical works. This is illustrated in Figure 2 where the results of simulations are directly compared with the results of TMS-EEG experiments both in the time domain and frequency domain to detect the presence of cortical bistability (slow waves and Off-periods). This analysis shows a fundamental qualitative correspondence between Case 2 of the model (Fig. 2*A*) and spontaneous as well as evoked patterns of electrophysiological activity found in humans across different conditions encompassing N2 sleep (Massimini et al., 2007; Fig. 2*B*), UWS patients (Rosanova et al., 2018; Fig. 2*C*), and perilesional area of stroke patients (Sarasso et al., 2020; Fig. 2*D*). In all these cases, TMS-evoked slow waves and Off-periods, as detected by a significant high-frequency (>20 Hz) power suppression, are reliably revealed by cortical perturbations, whereas they are rare in the spontaneous prestimulus activity. As a note, Figure 2 shows a qualitative agreement between Case 2 in the model and the experimental data. By choosing the model in Equation 1, we aimed to unveil the mechanisms behind cortical bistability through an extensive exploration of the model phenomenology. Given that the model describes the firing rates of a local neuronal population, which differ from the experimental data in both the kind of signal and the spatial scale involved, a quantitative comparison between simulated and experimental data were beyond the aim of the present work.

**Figure 3.**
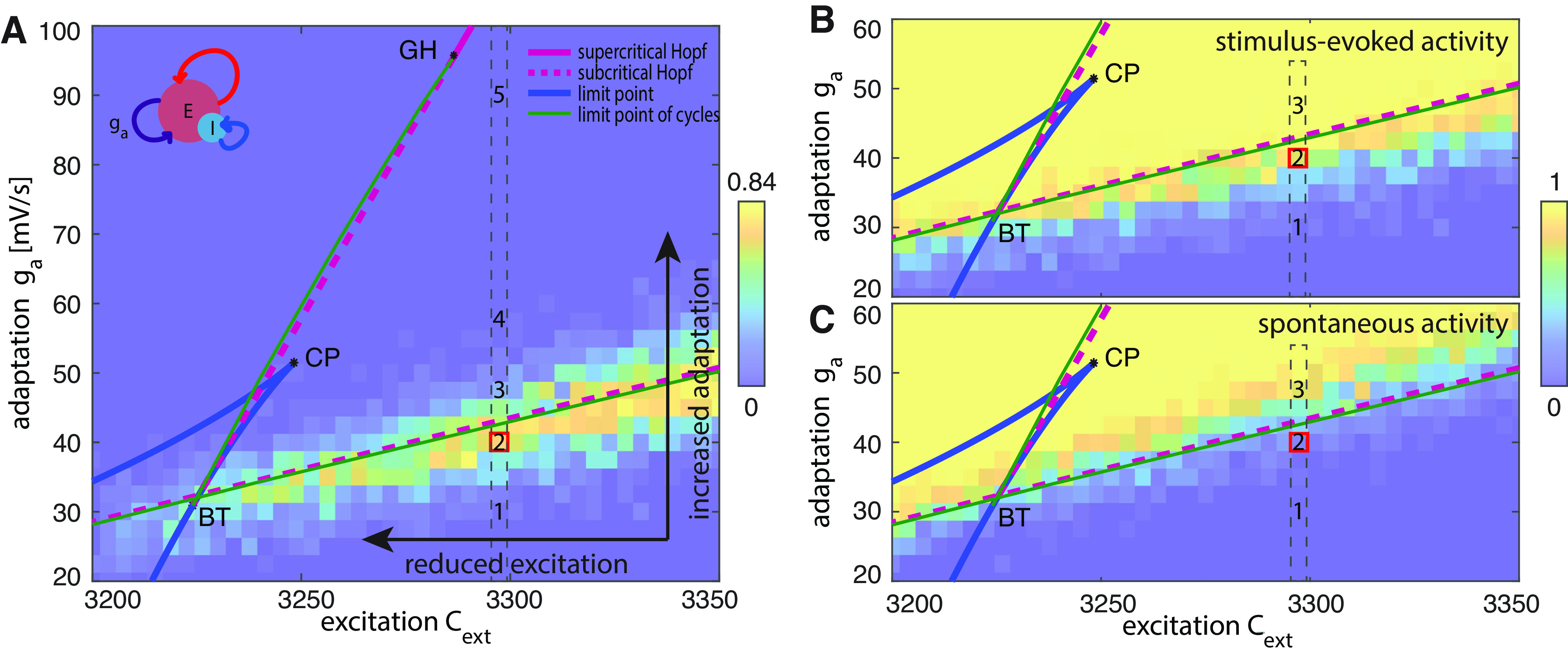
Off-periods can be better revealed by looking at stimulus-evoked activity than at spontaneous activity. Panel ***A*** shows the differences between the probability of evoking Off-periods compared with observing spontaneous Off-periods for each excitation-adaptation pair, superimposed on the bifurcation diagram of the population rate model shown in Figure 1. The differences between the probability of evoked Off-periods and spontaneous Off-periods for Cases 1–5 are as follows: Case 1: 0; Case 2: 0.7; case 3: 0.24; Case 4: 0; Case 5: 0. Panel ***B*** shows the fraction of stimulus-evoked Off-periods (Case 1: 0; Case 2: 0.76; Case 3: 1). Panel ***C*** presents the probability of Off-periods detected in the spontaneous dynamics (Case 1: 0; Case 2: 0.06; Case 3: 0.76). Panel ***A*** shows results for adaptation values spanning from 20 to 100 mV/s, while panels ***B*** and ***C*** focus on adaptation values between 20 and 60 mV/s. In all the panels, the probability of Off-periods has been computed by counting the number of trials, lasting 2 s each, that show Off-periods divided by the total number of trials (50).

Considering its prevalence in real data across different physiological and pathologic conditions, we asked whether such dissociation between spontaneous and stimulus-evoked activity is a peculiar feature of the specific level of excitation, or whether it can be generalized to a large interval of excitation levels. Figure 3 shows the difference in the frequency of Off-periods detected by analyzing the spontaneous and evoked activity for each excitation-adaptation pair. We found an area surrounding the low Hopf bifurcation curve, extending to ∼10% of the analyzed excitation-adaptation plane, characterized by a pronounced difference between evoked and spontaneous Off-periods. More specifically, despite a marked difference being present for all the excitation levels higher than $C_{E,ext}=3250$, a near-complete dissociation (∼84% of evoked trials detect Off-periods where the spontaneous trials do not show any) can be shown for very high excitation levels.

Given the shallow slope of the lower branch of the Hopf bifurcation, changes in adaptation are expected to have more profound effects on cortical bistability than shifts in the excitation level. However, substantial changes in excitation levels for a given adaptation (i.e., horizontal shifts toward the left in the bifurcation diagram) still result in changes in spontaneous and stimulus-evoked dynamics similar to those reported in Figure 1. Extended Data Figure 1-1 illustrates such cases, including the interesting instance (Case 2) characterized by an intermediate excitation level, showing the dissociation between spontaneous and stimulus-evoked dynamics.

### High adaptation level is associated with early breakdown of the causal interactions compared with low adaptation

**Figure 4.**
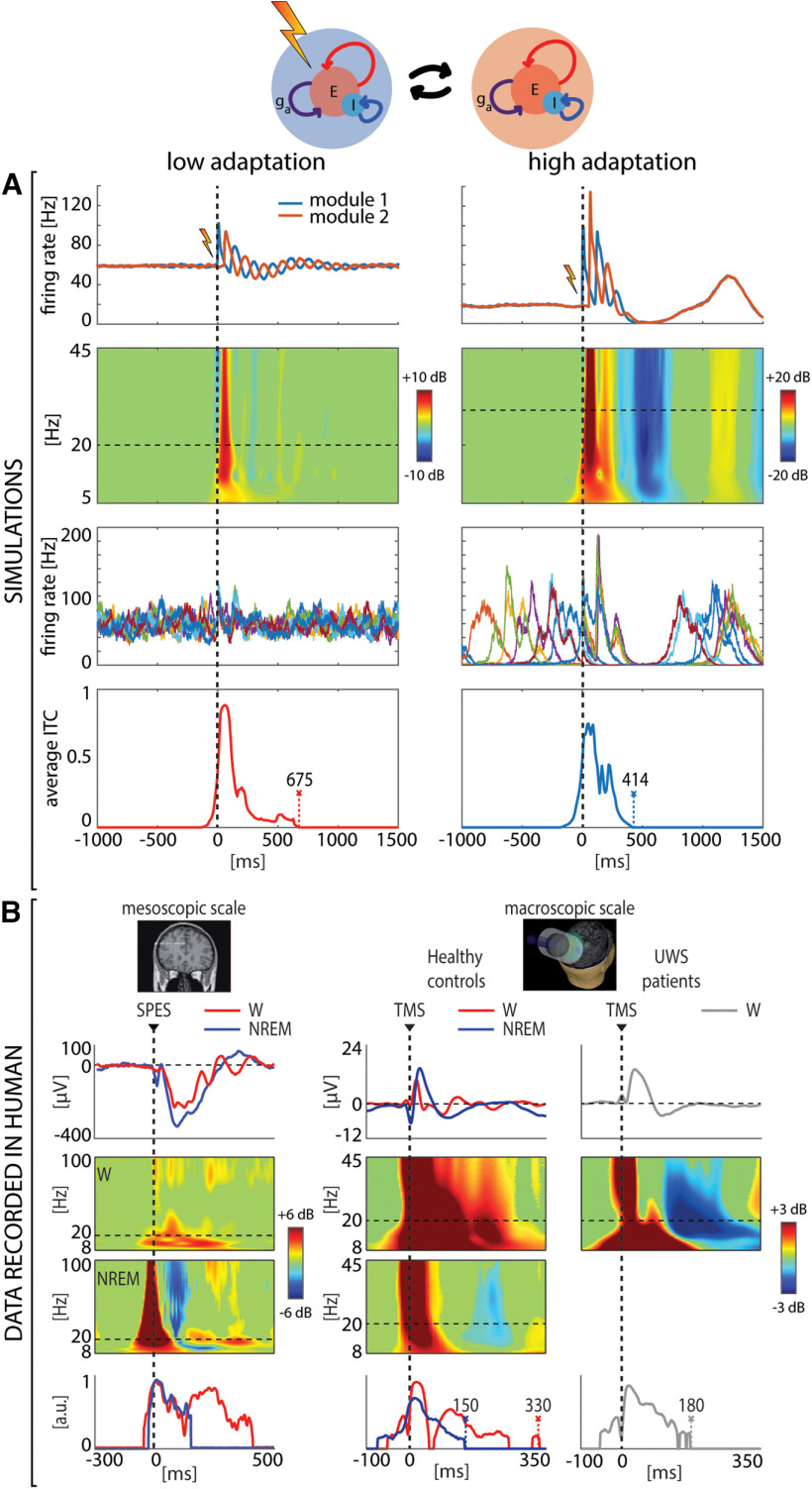
High adaptation level results in early breakdown of causal interactions compared with low adaptation level. ***A***, Simulated data of two coupled modules for low ( $g_{a}=48$ mV/s) and high ( $g_{a}=78$ mV/s) adaptation levels. First row, Firing rate activity averaged over 250 trials. A dashed vertical line (at *t* = 0) marks the occurrence of the external stimulus injected into module 1. Second row, Event-related spectral perturbation (ERSP) for module 2. Significance for bootstrap statistics is set at α < 0.005: absence of significant activation is colored in green, significant increases of power compared with baseline are represented in red, while significant power decreases are colored in blue. Third row, Eight firing rate traces of module 2. Fourth row, Averaged intertrial coherence (ITC) for frequencies between 5 and 30 Hz. ***B***, Data recorded in human. The first column shows results related to stereo-EEG with SPES during wakefulness (W-red) and NREM sleep (NREM-blue) re-edited from Pigorini et al. (2015). From top to bottom, average responses of a representative contact, event-related spectral perturbation (ERSP), and phase-locking factor (PLF) for frequencies higher than 8 Hz (details can be found in Pigorini et al., 2015). Second and third columns show results related to TMS-EEG re-edited from Rosanova et al. (2018), encompassing healthy wakefulness (W-red), healthy NREM sleep (NREM-blue), and UWS patients during wakefulness (gray W). As for stereo-EEG-SPES data, from top to bottom, average responses of the channel under the stimulator, event-related spectral perturbation (ERSP), and phase-locking factor (PLF) for frequencies higher than 8 Hz (details can be found in Rosanova et al., 2018).

Empirical studies employing perturbations in humans (Pigorini et al., 2015; Usami et al., 2015; Rosanova et al., 2018), rodents (Arena et al., 2021), and cortical slices (D’Andola et al., 2018) suggest that cortical bistability and the associated Off-periods can disrupt the emergence of sustained patterns of causal interactions among cortical neurons. We thus used the model to test whether changes in adaptation level can also affect casual interaction among groups of cortical neurons. To do that, we considered two cortical modules, instead of one as in previous sections, connected through reciprocal bidirectional connections. As in this new model composed of two interacting modules, a complete characterization of the bifurcations would require exceedingly high computational cost, we here focused on a representative scenario characterized by two extreme adaptation levels (Fig. 4*A*). Specifically, the low adaptation level parallels the case of wakefulness as seen in the previous section (Case 1; Fig. 1*B*), as opposed to the high adaptation level characterizing N3 sleep or deep anesthesia (Case 4; Fig. 1*B*). Under conditions of low adaptation, cortical perturbations elicited reverberant interactions between the two modules as reflected by multiple, recurrent and coherent oscillations of evoked activity. Conversely, under conditions of high adaptation, the interplay between the two simulated groups of cortical neurons was short-lasting resulting in only a few oscillations followed by a prominent Off-period, as marked by the suppression of high-frequency power, similar to that observed in sleeping and brain-injured humans.

In real-life experiments, the impact of the Off-period on the ability of cortical circuits to sustain causal interactions is assessed by quantifying the duration of the deterministic effects of the perturbation in the phase domain. A key empirical finding is that Off-periods not only disrupt deterministic interaction because of the associated suppression of power but that they also scramble the subsequent phase of the signal when neurons resume firing. Such stochastic reboot is characterized by oscillations with high power but low phase-locking or intertrial coherence following the Off-period. We thus performed the same analysis in the model by computing the intertrial coherence (ITC) averaged across frequencies between 5 and 30 Hz. As shown in Figure 4*A*, the Off-period occurring under conditions of high adaptation was followed by a broad-band resumption of power which was however nondeterministic as indicated by a concomitant absence of significant ITC. The primary sources of such stochasticity are (1) the variable level of fatigue accumulated by the network at the stimulation time and (2) the activity fluctuations because of the finite-size of the neuronal population determining the escape time from the metastable inactive state (Off-period). Notably, in the case of low adaptation, significant deterministic interactions were not curtailed by the Off-period and were longer-lasting. As illustrated in Figure 4*B*, these model predictions resulting from the manipulation of the adaptation levels match the power and phase modulations observed in experiments in which TMS or intracranial electrical stimulations are applied in different global brain states, such as during wakefulness, deep NREM sleep and in UWS patients.

## Discussion

By exploiting a spiking network model endowed with activity-dependent adaptation, the present work provides a principled mechanistic explanation for empirical results revealing common alterations of cortical responsiveness in sleeping healthy subjects, and in awake patients with multifocal and focal brain injuries. Specifically, (1) we link changes in cortical reactivity to the presence of underlying activity-dependent adaptation mechanisms, above and beyond spontaneous dynamics, (2) we explain the role of perturbations in revealing adaptation mechanisms, and (3) we expound on the impact of adaptation in disrupting causal interactions among cortical modules.

The first implication of this work is that systematically varying the relationships between excitation and adaptation levels within a formal bifurcation analysis reproduces basic patterns of cortical reactivity to direct perturbations observed in humans. As shown in Figure 1*A*,*B*, increasing levels of adaptation and/or decreasing the excitation engenders a condition whereby cortical circuits react to a direct perturbation with an initial activation followed by an Off-period, yielding responses similar to those found during sleep and in awake brain-injured patients. This finding offers important elements for interpreting macroscale empirical data in terms of neuronal mechanisms.

In this framework, the Off-periods observed after cortical stimulation in sleeping humans can be ascribed to adaptation mechanisms because of slow negative (i.e., inhibitory) feedback produced by calcium-gated and sodium-gated potassium currents (Sanchez-Vives and McCormick, 2000; Sanchez-Vives et al., 2010), which are enhanced by decreased levels of neuromodulation from brainstem activating systems (McCormick, 1992). Notably, the present simulation results closely match the changes in reactivity to electrical stimulation observed in cortical slices when modulating potassium currents by applying carbachol and norepinephrine (D’Andola et al., 2018). Recent studies have also pointed to a putative role of active inhibition in conditioning the onset and duration of the Off-period (Funk et al., 2017; Zucca et al., 2017). As adaptation in the present model encompasses self-inhibition, the role of local inhibitory neurons in shaping the changes in cortical responses observed during sleep is not incompatible with the present results.

Interpreting the empirical results obtained in brain-injured and stroke patients within the theoretical framework of the bifurcation diagram discloses a more complex and interesting landscape. Indeed, brain lesions can cause global or local alterations of adaptation and excitation through different mechanisms, even in the context of preserved arousal, as assessed by eyes opening. For example, in some UWS patients, lesions, compressions, or displacements of brainstem activating systems as well as a critical load of damage to ascending fibers in subcortical white matter may enhance potassium currents (Steriade et al., 1993; Edlow et al., 2012), which corresponds to an upward shift in the bifurcation diagram. In other cases, cortical injuries and white matter lesions can engender a state of corticocortical disfacilitation by affecting the excitation term (Takahashi et al., 1981; Lemieux et al., 2014; Rocchi et al., 2022). In this case, the resulting loss of lateral and long-range excitatory input would act by producing shifts on the horizontal axis toward the left, to a point where the probability of evoking an Off-period in an awake patient becomes higher. An interesting implication of the diagram, especially considering the slope of the lower branch of the bifurcation (Figs. 1*A*, 3), is that changes in adaptation are expected to have more dramatic effects on cortical bistability as compared with changes in the excitation level. This prediction finds empirical confirmation in cortical slices, an extreme model of cortical injury, whereby reducing adaptation by application of carbachol and norepinephrine is more effective in recovering wake-like responses than increasing excitation by application of kainate (D’Andola et al., 2018). In real-life conditions, however, the two mechanisms (adaptation and excitation) are not mutually exclusive and may have different relative weights depending on the type/combination of injuries. In fact, they may both concur in bringing residual cortical circuits into a state in which cortical sleep-like Off-periods are generated during wakefulness, as observed in stroke (Sarasso et al., 2020) and UWS (Rosanova et al., 2018) patients.

The second implication of this work lies in the dissociation between observational and perturbational approaches in their ability to unveil the presence of cortical bistability and Off-periods, and bears relevance for the assessment of the aftermath of brain injury. Such dissociation is represented by the area in the excitation-adaptation bifurcation diagram identified in Figure 3, in which the responses to perturbations more reliably show Off-periods as compared with spontaneous activity. This portion of the diagram encompasses a relatively small space of the overall dynamics (ranging from wake-like activity to patterns resembling burst suppression) but is extremely relevant in real-life conditions. Indeed, cortical perturbations, either electrical or magnetic, are often capable of evoking clear-cut Off-periods, which are not otherwise present in spontaneous activity not only during N2 sleep but also in many stroke and traumatic brain-injury patients (Rosanova et al., 2018; Sarasso et al., 2020). This is a critical working point along with the sleep-wake transition, in which cortical dynamics are intrinsically unstable as two global activity modes compete (Parrino et al., 2012; Tort-Colet et al., 2021). From a therapeutic perspective, this offers a window of opportunity as small physical or pharmacological perturbation of the network parameters can induce dramatic changes in the brain global behavior (Deco et al., 2019). Thus, relating empirical findings to this region of the bifurcation diagram is important for two reasons. First, it suggests that a significant number of patients may lay in a state where the input-output properties of cortical circuits are altered because of a critical, albeit potentially reversible, shift in adaptation (and/or excitation). Second, it shows that, because of its inherent activity-dependent nature, this state of affairs can be better revealed, above and beyond the observation of spontaneous dynamics, by challenging cortical circuits with direct stimulation.

The third result of this theoretical and computational work is that altered levels of adaptation can have profound effects on the capability of cortical neurons to engage in reciprocal interactions, as indicated by the complex set of effects observed when changing adaptation in two reciprocally connected cortical modules without modulating their connectivity strength. Under conditions of low adaptation, the two simulated modules engage in a long-lasting series of feed-forward and feed-back interactions leading to multiple waves of activity time-locked to the stimulus, resembling the general pattern of responsiveness found in cortical slices under carbachol and norepinephrine (D’Andola et al., 2018) and in healthy awake subjects (Massimini et al., 2005). For high adaptation, this deterministic pattern of interaction is drastically curtailed; not only Off-periods temporarily obliterate activity, but they also disrupt phase-locking to the stimulus once activity resumes. This peculiar condition, whereby high power is associated with minimal levels of phase-locking to the stimulus, is strikingly similar to the pattern found in multiscale empirical measurements ranging from cortical slices (D’Andola et al., 2018) and anesthetized rodents (Arena et al., 2021) to human intracranial and extracranial measurements during NREM sleep and after severe brain injury (Pigorini et al., 2015; Rosanova et al., 2018). Notably, in the brain of patients, Off-periods and the ensuing disruption of causal interactions are empirically associated with low values of whole-brain complexity and with loss of consciousness (Rosanova et al., 2018). Perhaps more importantly, the progressive disappearance of evoked Off-periods is associated with recovery from disorders of consciousness (Rosanova et al., 2018) and stroke (Tscherpel et al., 2020). In light of the present theoretical framework, this clinical evolution would correspond to the descending trajectory represented in the bifurcation diagram of Figure 1*A*, with potential implications for stratification, follow-up, and rehabilitation in the aftermath of brain injury. For example, detecting cortical bistability by perturbations in a stroke patient points to the presence of functional disruption, adding to the structural damage, and suggests that neuromodulation or pharmacological treatment should aim at reducing adaptation mechanisms and/or strengthening local excitation until the occurrence of evoked Off-period is minimized.

Clearly, the present theoretical framework only represents a first stepping stone on which more realistic and complex models can be built, such as those incorporating the topological organization of cortical networks (Capone et al., 2019; Barbero-Castillo et al., 2021; Pazienti et al., 2022). To be comprehensive such models should also include other subcortical structures like the thalamus, whose input is known to play an important role in shaping Off-periods (van Wijngaarden et al., 2016; Zucca et al., 2019). Besides, the present work only provides a minimal account, limited to the proof of principle of two connected modules of the effects of adaptation on corticocortical interactions. Hence, a fundamental development will consist in embedding the present framework within a large-scale, connectome-based simulation, such as the “The Virtual Brain,” encompassing a multitude of interacting modules (Sanz Leon et al., 2013; Kringelbach et al., 2020; Goldman et al., 2022; Schirner et al., 2022). This would offer a tool to better understand the effects of local intrusions of cortical bistability within the awake brain, such as those occurring after sleep deprivation (local sleep; Hung et al., 2013; Sarasso et al., 2014; Bernardi et al., 2015; Nir et al., 2017) as well as those occurring when adaptation and excitation are altered in a regional-specific manner by focal and multifocal structural lesions.

Several computational models have been proposed in the last years to describe in detail the effect of electrical stimulations on brain circuits (Bai et al., 2013; McIntyre and Foutz, 2013; Seo and Jun, 2017; Farokhniaee and McIntyre, 2019; Komarov et al., 2019). Despite some lack of biophysical details in our model network, we implemented a parsimonious description of the general effect of physical (magnetic or electric) cortical perturbations. Indeed, we considered both electrical and magnetic stimulations as capable of inducing intraparenchymal electric fields (Nieminen et al., 2015; Rahman et al., 2015; Laakso et al., 2018). Such local and exogenous fields are known to modulate neuronal excitability inducing a polarization of the membrane potential of pyramidal cells with orientation parallel to the field vector (Bikson et al., 2004; Radman et al., 2009). Based on this experimental and theoretical evidence, we eventually modeled TMS and electrical stimulations as a transient change in the input currents received by excitatory (i.e., pyramidal) cells in our spiking neuron network, leading in turn to a depolarization of their membrane potential. Remarkably, such a simple model allowed us to qualitatively reproduce the evoked responses shown in experimental works and to reveal that the adaptation level in the network strongly shapes those evoked responses. In the future, our network will benefit from increased biophysical details to differentiate between electrical and magnetic stimulations, and to explore the fine grained, intracolumnar events underlying activity-dependent adaptation.

10.1523/ENEURO.0435-22.2023.ed1 Extended Data 1 Code accessibility statement. The included computer code is in two parts. First is a MATLAB script entitled “SpikingNeuronNetwork_Perseo.m” to simulate our networks through Perseo, which is open-source software available at https://github.com/mauriziomattia/Perseus. Also, a Python code entitled “SpikingNeuronNetwork_nest.py” allows the simulation of the spiking neuron network presented in this work using the NEST. This Python code provides an explicit and detailed implementation of the spiking neuron network we modeled. The second part is made of two MATLAB scripts entitled “main_1Module.m” and “main_2Modules.m.” These scripts were used to present and analyze the firing rate time series of the single-module and two-module spiking neuron networks having specific excitation and adaptation levels. Download Extended Data 1, ZIP file.

## Synthesis

Reviewing Editor: Christoph Michel, Universite de Geneve Decisions are customarily a result of the Reviewing Editor and the peer reviewers coming together and discussing their recommendations until a consensus is reached. When revisions are invited, a fact-based synthesis statement explaining their decision and outlining what is needed to prepare a revision will be listed below. The following reviewer(s) agreed to reveal their identity: Viktor Jirsa.

Both reviewers have several methodological concerns that need to be addressed. Also, the existing literature in the field needs to be better introduced and discussed. One reviewer suggest to include the supplementary figure in the main text. Please consider.

The detailed comments of the reviewers are added here:

Reviewer 1:

The authors discuss fundamental properties of the local responses elicited in

humans by direct cortical stimulation. Model simulations shall address these behaviors and are replicated by systematically varying the relationships between adaptation strength and excitation level in the network. Bifurcation analyses and simulations of perturbations are presented to mimic features observed experimentally.

In the introduction, the authors provide a thorough discussion of the literature of up and down states as known from physiological imaging and corresponding mean field modeling. The discussion is poor in terms of modeling of mechanisms underlying stimulation. The latter is large, examples of studying stimulation mechanisms in population models could be from the groups of Bazhenov, McIntyre, Jirsa, Terman, just to name a few basic contributors. This is definitely a short coming.

Examples:

Komarov M, Malerba P, Golden R, Nunez P, Halgren E, Bazhenov M. Selective recruitment of

cortical neurons by electrical stimulation. PLOS Comput Biol 2019; 15: e1007277.

Farokhniaee A, McIntyre CC. Theoretical principles of deep brain stimulation induced synaptic

suppression. Brain Stimul 2019; 12: 1402-9.

In the methods section, the approaches are defined. The model representations are satisfactory and include 1) Spiking neuron network 2) Neural populations, mean field ;

For the simulation: Why was this simulator for the spiking neurons chosen and not another more one, maybe more standard such as NEST? What algorithm was used for the mean field?

For both: Describe better the origin of “a”, where does the variable “a” come from, what is its motivation, etc?

In the Results:

Figure 1 is a central figure, but needs help. Fig 1A has been made based on the mean field equations, using a standard bifurcation package based on continuation. This is ok. From Page 11, it is suggested that Fig 1B is based on the detailed Integrate and fire neurons, this is not evident from the caption. In the Caption, identify the acronyms. The figure should show as a minimum the corresponding simulations of the mean fields, not just the spiking neuron network. The right hand side of the figure 1B shows trajectories in phase space. Show the equivalent for the mean fields, identify the flows in state space and identify the attractors in state space. Make the correspondence between simulated spiking neural networks and mean fields. Verify the attractor structure. All these are standard expectations one would have when juxtaposing the micro and macro models.

Some more technical: Explain limit points as saddle-node bifurcation of periodic orbits and identify the abbreviations, etc (LPC stands for ‘Limit Point of Cycles’).

The rightmost part of the traces in Fig. 1B (left column) shows 6 seconds of the stimulus-evoked activity for a given excitation value and five different adaptation levels. This needs more detail. With my comments and suggested changes above, I would recommend to split Figure 1 in two figures, given its importance for the paper.

The discussion is ok, but will depend a lot on the above changes. As such, in the current version, it is not sufficiently strongly supported by evidence in the simulations.

Reviewer 2:

The article investigates an important topic: trying to understand differences in evoked brain dynamics in different states. The choice of models is sound, as is the presentation on the whole. The results are novel. The analysis of perturbations is qualitative and not particularly extensive, but is sufficient for publication. I have a few comments, predominantly on the presentation of methods and results, that should be addressed before publication:

additional clarification of the methods would be helpful for the general reader, and to make the article a little more self contained:

- could you please point explicitly to a reference for the mean and variance of the input current, as this was difficult to locate in the references provided.

- for consistency, please write tauv_alpha etc in the sentence “the evolution of the firing rate v_alpha..”. In this same sentence: networks -> network.

- could you please give a brief explanation of / justification for the equation tau_v = -v + phi(v) (in addition to the references provided, so the uninitiated reader does not have to dig through those).

- please provide further justification for why tau_I can go to zero

- I was unconvinced of the shifting of the bifurcation diagram - please explain why it is important to do this rather than showing the raw results, or else just show the raw results.

- it would be thorough to show time series from the grey region in figure 1, especially since you referred to this region of the bifurcation diagram with respect to modelling the cognitive function of working memory. Similarly, can you please show the dynamics of the population model.

- Can you also expand on the comparison of dynamics of the model vs the cognitive function of working memory - specifically which features of the data are captured by the model?

p12: can you provide a reference for having modelled perturbation specifically as a 1ms pulse. Moreover, I didnt see a specific parameterisation of the stimulus input current in the model - please provide this.

p12: it is claimed that the duration of off periods increases with the increase in the adaptation parameter. Certainly for examples 2,3 and 4 it seems that the off period is the same duration. Please clarify this observation.

Please comment on why the time scales in the simulated stimulation are different to the data (as presented in the ERSP)

The context for the results in Fig3 is that in the data and the simulation of figure 2 there are *no* off periods in the spontaneous activity, but there are in the stimulated epochs. With this in mind, the description of Fig 3 is quite subjective, referring to “pronounced” differences between ‘spontaneous off’ and ‘stimulus induced off’ periods. It seems to me that it would be more appropriate to show values of 1 in figure 3, rather than the full range of [0,1], as the former would be more consistent with the idea that the spontaneous activity should not contain any off periods. It seems that the regions of parameter space for which the spontaneous activity has *no* off periods would infact be very small?

Please provide details of the parameter values used and coupling function for the models of figure 4.
