# Supplementary figures and images for: Adaptation Shapes Local Cortical Reactivity: From Bifurcation Diagram and Simulations to Human Physiological and Pathological Responses
Source: eNeuro. 2023 Jul 24;10(7):ENEURO.0435-22.2023. doi: 10.1523/ENEURO.0435-22.2023 (PMC10368205; doi:10.1523/ENEURO.0435-22.2023)

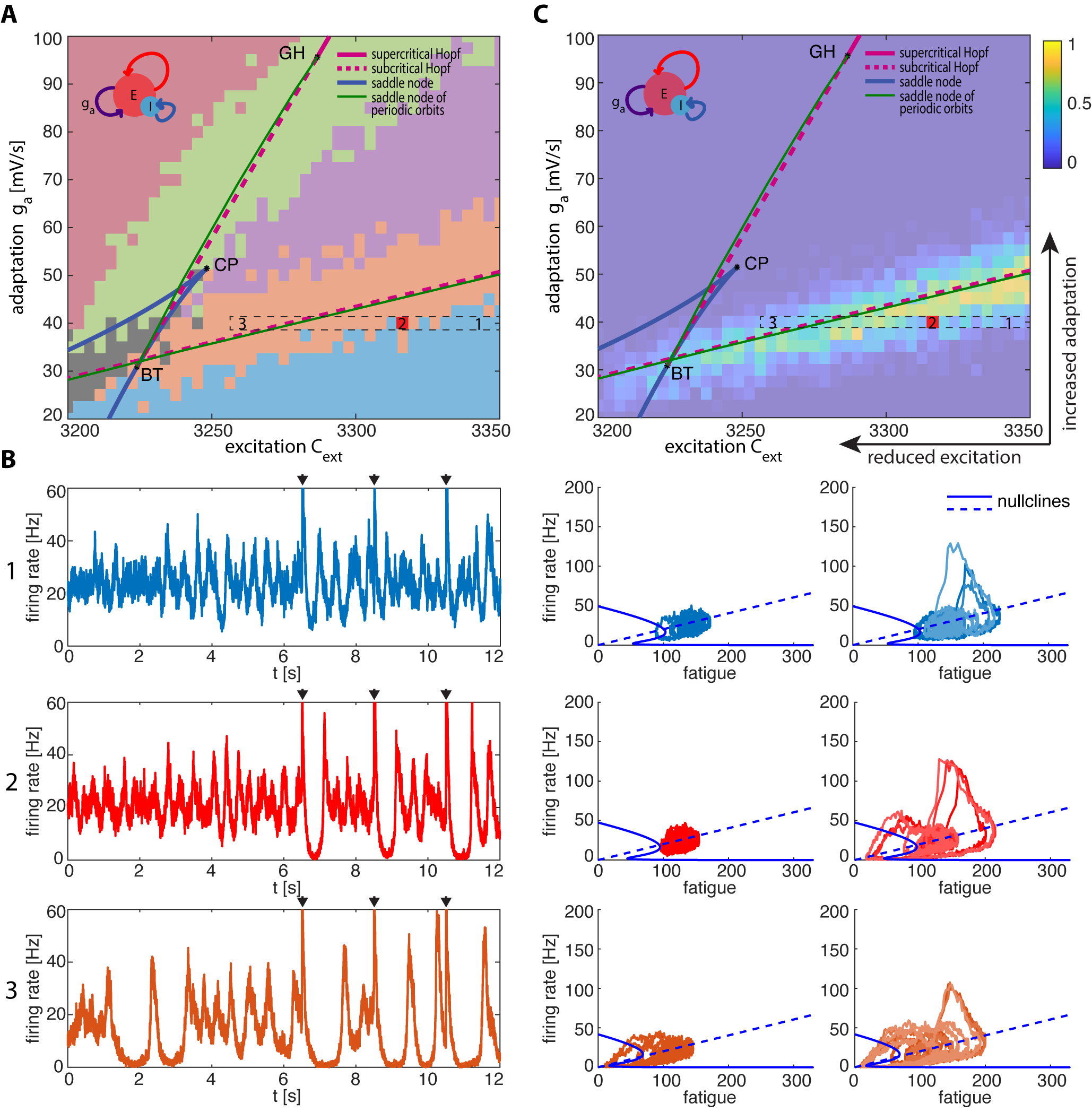

Supplement: Extended Data Figure 1-1 — Bifurcation analysis of the population rate model and dynamical regimes of the spiking neuron network (as in Fig. 1) for a fixed adaptation level (ga=42.5 mV/s) and different excitation levels (3342.5, 3320, 3248.75). A, C, As in Figures 1 and 3, respectively. In panel C, the differences between the probability of evoked Off-periods and spontaneous Off-periods for Cases 1–3 are as follows: Case 1: 0.08; Case 2: 0.46; Case 3: 0.14. B, Spontaneous and stimulus-evoked signals (using the same color coding as in A) for a fixed level of adaptation. Left column, Time series encompassing both spontaneous activity (up to 6000 time steps) and stimulus-evoked activity (remaining interval). Black triangles indicate the occurrence of the stimulation. Central column, Spontaneous activity as a function of fatigue. Right column, Three superimposed orbits due to perturbations as a function of the fatigue. Download Figure 1-1, TIF file. [file enu-eN-NWR-0435-22-s02.tif]
